# The Genome of the Chicken DT40 Bursal Lymphoma Cell Line

**DOI:** 10.1534/g3.114.013482

**Published:** 2014-09-15

**Authors:** János Molnár, Ádám Póti, Orsolya Pipek, Marcin Krzystanek, Nnennaya Kanu, Charles Swanton, Gábor E. Tusnády, Zoltan Szallasi, István Csabai, Dávid Szüts

**Affiliations:** * Institute of Enzymology, Research Centre for Natural Sciences, Hungarian Academy of Sciences, H-1117 Budapest, Hungary; † Department of Physics of Complex Systems, Eötvös Loránd University, H-1117 Budapest, Hungary; ‡ Center for Biological Sequence Analysis, Department of Systems Biology, Technical University of Denmark, DK-2800 Lyngby, Denmark; § Cancer Research UK London Research Institute, London, WCA2 3PX, United Kingdom; ** Children's Hospital Informatics Program at the Harvard-Massachusetts Institutes of Technology Division of Health Sciences and Technology (CHIP@HST), Harvard Medical School, Boston, MA 02115

**Keywords:** DT40, gene targeting, tumor suppressor gene, chicken genome, single nucleotide polymorphism

## Abstract

The chicken DT40 cell line is a widely used model system in the study of multiple cellular processes due to the efficiency of homologous gene targeting. The cell line was derived from a bursal lymphoma induced by avian leukosis virus infection. In this study we characterized the genome of the cell line using whole genome shotgun sequencing and single nucleotide polymorphism array hybridization. The results indicate that wild-type DT40 has a relatively normal karyotype, except for whole chromosome copy number gains, and no karyotype variability within stocks. In a comparison to two domestic chicken genomes and the *Gallus gallus* reference genome, we found no unique mutational processes shaping the DT40 genome except for a mild increase in insertion and deletion events, particularly deletions at tandem repeats. We mapped coding sequence mutations that are unique to the DT40 genome; mutations inactivating the *PIK3R1* and *ATRX* genes likely contributed to the oncogenic transformation. In addition to a known avian leukosis virus integration in the *MYC* gene, we detected further integration sites that are likely to de-regulate gene expression. The new findings support the hypothesis that DT40 is a typical transformed cell line with a relatively intact genome; therefore, it is well-suited to the role of a model system for DNA repair and related processes. The sequence data generated by this study, including a searchable *de novo* genome assembly and annotated lists of mutated genes, will support future research using this cell line.

The DT40 cell line has been used as an important model system in the study of DNA repair and immunoglobulin diversification for more than two decades. The cell line is derived from a bursal lymphoma of a female domestic layer chicken infected with avian leukosis virus (ALV) (Baba and Humphries 1984; Baba *et al.* 1985). Efficient homologous gene targeting in DT40 makes gene disruptions and sequence manipulations feasible; therefore, the cell line is uniquely suited to genetic studies (Buerstedde and Takeda 1991). Gene targeting is preferable to gene silencing in many experimental applications due to the completeness of its effect on protein expression, which is especially important when the genetic interactions of several factors are investigated. The use of sequence-specific nucleases such as TALE nucleases and the CRISPR system has recently presented an alternative method of gene manipulation in cell lines (Cermak *et al.* 2011; Ran *et al.* 2013); however, the simplicity of the homology-based targeting coupled to the predictability of the outcome and the traceability of off-target integration will support the future use of DT40 genetics.

The design and interpretation of gene targeting experiments are facilitated by the detailed characterization of the genome of the host cell line. In general, DT40 cells have been reported to display a stable karyotype (Sonoda *et al.* 1998). Although further karyotype analysis detected variation in the number of macrochromosomes between and within DT40 cultures (Chang and Delany 2004), the relative normality and stability of the karyotype of this cancer cell line are significant advantages for its use in genetic experiments.

The design of gene targeting constructs has been much more rapid since the publication of the draft chicken genome in 2004 (International Chicken Genome Sequencing Consortium 2004). The latest genome build (Gallus_gallus-4.0) was released in 2011, and it covers 1.072 × 10^9^ base pairs on 29 of the 37 autosomes, the sex chromosomes W and Z, and further unplaced contigs. A limitation of using this reference genome for DT40-based research is the poor assembly of the microchromosomes. Several of these are entirely absent from the reference genome, and the higher microchromosomal gene densities (Smith *et al.* 2000) increase the probability that genes of interest are on unplaced contigs. A further limitation is the sequence divergence between the reference red jungle fowl and domestic breeds from which DT40 is derived. Indeed, 2.9 million to 4.6 million single nucleotide variations (SNVs) were identified in each of 8 domestic chicken breeds using ABI SOLiD sequencing with 3.32–5.53× coverage, and 6.0 and 5.8 million SNVs were found by Illumina sequencing with 23–25× coverage in two further domestic breeds, the Taiwanese L2 and the Silkie breeds (Rubin *et al.* 2010; Fan *et al.* 2013), in comparison with the Gallus gallus reference genome. Isogenic targeting DNA is generally used for homologous targeting as nonisogenic DNA greatly reduces targeting efficiency (te Riele *et al.* 1992). Prior knowledge of the cell line–specific genomic DNA sequence therefore helps the planning and assembly of the gene targeting constructs, especially if homology arms are produced by gene synthesis.

Experiments on cell lines that serve as biological model systems should be interpreted in light of specific properties of the cell line. Exome sequencing of a large number of human cancer cell lines has identified mutations in known cancer genes (Barretina *et al.* 2012; Garnett *et al.* 2012). However, these datasets do not contain the sequence of all genes or that of intergenic regions. Full genomic sequence data have been obtained for some cancer cell lines, for example, HeLa, in which coordinated genome sequencing and expression analyses were used to characterize the cell line (Adey *et al.* 2013; Landry *et al.* 2013). Similarly, annotated whole genome sequence data will help the future use of DT40 as one of the best-established genetic model systems.

In this study we set out to analyze the genomic sequence of the DT40 cell line at high coverage and identify SNVs and insertions/deletions (indels) in comparison with the *Gallus gallus* reference genome. We compared the SNV and indel dataset with those of published domestic chicken breeds to determine whether cell line–specific mutagenic processes operate in DT40. The analysis of copy number changes in the sequenced clone and in further wild-type DT40 clones suggests a relative stability of the DT40 karyotype. The raw sequence, a *de novo* genome assembly, and the results of the sequence analysis are made available as a resource to the DT40 research community.

## Materials and Methods

### Cell culture and sample preparation

DT40 wild-type cell line stocks originally sourced from the Institute of Animal Health (now Pirbright Institute, UK) were obtained from the laboratory of Dr Julian E. Sale, MRC Laboratory of Molecular Biology, Cambridge, UK. Cells were grown at 37° under 5% CO_2_ in RPMI-1640 medium supplemented with 7% fetal bovine serum, 3% chicken serum, 50 μM 2-mercaptoethanol, and penicillin/streptomycin. Single cell clones were isolated and grown prior to sample preparation. Genomic DNA for both SNP array analysis and DNA sequencing was prepared using the Gentra Puregene Cell Kit (Qiagen). The sample preparation for the SNP array analysis took place previously, and the cell clones were frozen in 90% fetal bovine serum plus 10% DMSO, stored in liquid nitrogen, and re-thawed prior to the preparation of the DNA sequencing sample.

### DNA sequencing and SNP array analysis

Libraries for next-generation sequencing were prepared using the NEBNext DNA Library Prep kit (New England Biolabs) with the omission of the PCR amplification step; 100 nt paired-end sequencing was performed on an Illumina HiSeq2000 instrument (Genomic Sequencing and Analysis Facility, The University of Texas at Austin). SNP array hybridization was performed at DNA Landmarks (St-Jean-sur-Richelieu, Quebec, Canada) using a 60,000-sample chicken SNP chip developed by Illumina Inc. for the GWMAS Consortium (Groenen *et al.* 2011).

### Data analysis

The reads were aligned to the chicken (*Gallus gallus*) reference sequence Galgal4.73, which was downloaded from Ensembl (Flicek *et al.* 2014). The alignment was made using the Burrows-Wheeler Alignment Tool (BWA, version 0.7.5a-r405) (Li and Durbin 2009). The reference sequence was indexed with the BWT-SW algorithm, which is recommended in the case of large genomes. The alignments of paired-end reads were generated with the *aln* and *sampe* algorithms. The generated alignment files were examined for general statistics, *e.g.*, number of mapped reads, coverage, insert-size distribution using the *sam-stats* program from the *ea-utils* package, and custom scripts (Aronesty 2011). Short genetic variants were identified with SAMtools (version 0.1.18 r982:295) (Li *et al.* 2009). In the pipeline, we used the options *mpileup-E-D-S-u* and *bcftools view-bvcg*. The variant calling format files were compressed with *bgzip* and indexed with *tabix* tools. The detected SNVs and indels were divided into distinct files and analyzed separately. SNVs were filtered with VCFtools applying default filters, except that coverage must be at least 3 and the value of the quality field must be at least 30 (*vcf-annotate -f +/Q = 30/d = 3 -H*) (Danecek *et al.* 2011). Short genetic variants (SNVs and indels) were annotated with ANNOVAR and CooVAR annotation tools using the Ensembl73 gene annotation file (Wang *et al.* 2010; Vergara *et al.* 2012). In the validation process, our SNV dataset was compared with the known chicken SNPs downloaded from Ensembl using the BEDtools *intersectBed* program (Quinlan and Hall 2010). We identified shared and unique SNP variants between DT40 and the L2 and Silkie breeds using BEDtools *multiintersectBed* program. Short indels were also identified with VarScan 2 (Koboldt *et al.* 2012) to confirm the data obtained with SAMtools. To detect LOH regions, we first counted the number of SNVs in 100-kb sequence blocks by VCFtools SNPdensity module, and then we selected those blocks where the number of homozygous SNVs was at least 10-times greater than the number of heterozygous SNVs and the number of homozygous SNVs was more than 50. CNV analysis was performed using R version 3.1.0 with Sequenza package version 2.0.1, available from CRAN, and using a protocol for missing normal sample (Favero *et al.* 2014). *De novo* genome assembly was performed by the Ray genome assembler, with k-mer size 31 and with the recommended options (Boisvert *et al.* 2010). All calculations and software tools were run on a Supermicro HPC cluster, with 256 CPU cores and 1TB RAM. The data analysis scripts are included as Supporting Information, File S1.

## Results

### Whole genome sequencing

**Figure 1:**
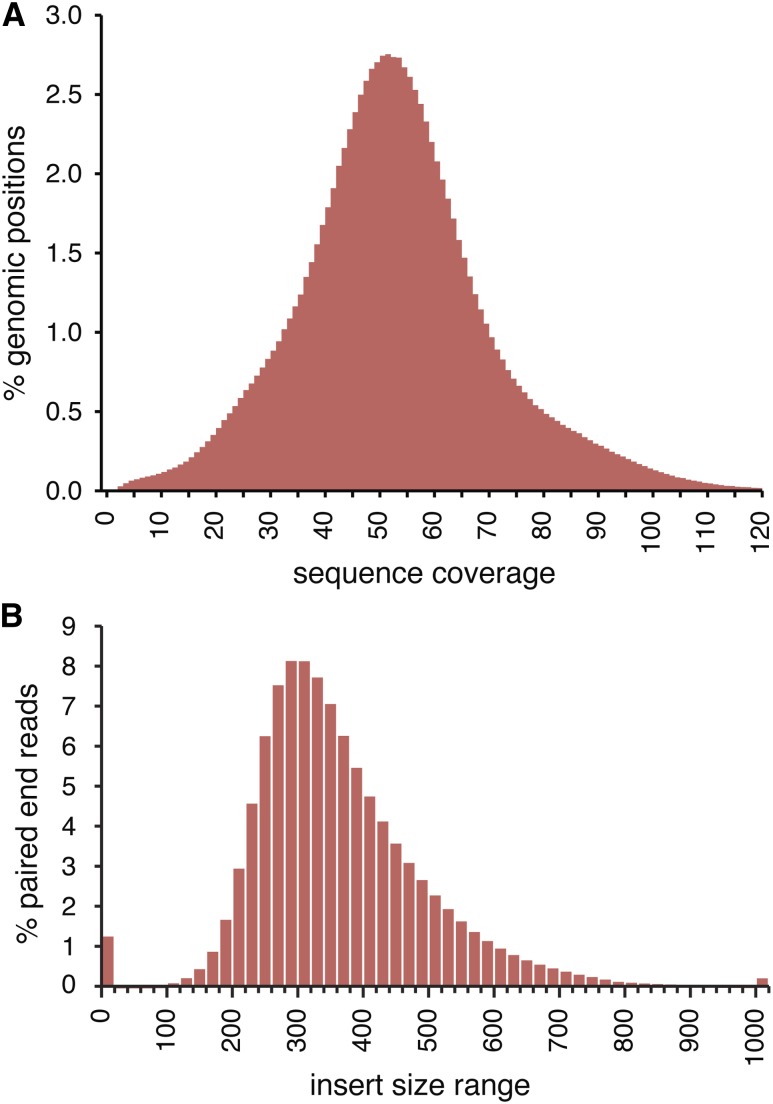
Whole genome sequencing of the DT40 cell line. (A) Histogram of the sequencing coverage across all positions of the *Gallus gallus* reference genome; 52× coverage is the most common. (B) Size distribution of the insert sequences of 100 nucleotide paired-end reads. The value 0 refers to unpaired reads, and the value more than 1000 includes all read pairs separated by more than 1000 base pairs.

A wild-type DT40 stock was chosen for analysis that was originally sourced from the Institute of Animal Health (WT-IAH) and has been extensively used in the Sale laboratory for DNA repair studies (Simpson and Sale 2003; Szuts *et al.* 2006). Genomic DNA was prepared from a single cell clone of this stock and sequenced on an Illumina HiSeq2000 instrument; 6.35×10^9^ 100 base pair reads were obtained with paired-end sequencing, of which 92.8% could be aligned to the Galgal4.73 reference genome, and 5.950×10^10^ base pairs were mapped over the 1.072×10^9^ base pair reference genome at 55× overall mean coverage, with a peak at 52× (Figure 1A). The mean insert size of the pairs was 353 (Figure 1B). A *de novo* assembly was also performed to aid the search for sequences not present in the reference genome. The assembly covered 9.802×10^8^ bp with contigs over 500 bp at an N50 value of 9801 bp, and 9.986×10^8^ bp with scaffolds over 500 bp at an N50 value of 28,885 bp (Table S1).

### SNVs and indels

We detected 6,251,553 SNVs in the DT40 genome, of which 3,320,786 are homozygous; 68% of these SNVs were already reported in dbSNP, which contains data from two sequencing efforts of domestic chicken breeds (Rubin *et al.* 2010; Fan *et al.* 2013). Because the genome of the original animal the DT40 cell line is derived from is not available, we cannot tell which of these mutations arose in the cell line after transformation. To explore this issue, we compared the DT40 genome with the genomes of two domestic breeds that were obtained with the same sequencing technology with high coverage (23–25×), the Taiwanese L2 and the Silkie breeds (Fan *et al.* 2013). Subjecting the raw sequence data to the identical analysis, we found SNV numbers in these samples that were very similar to those in DT40. In each case this corresponds to 5.8 to 5.9 SNVs per kilobase, similar to values reported in a number of chicken breeds (Rubin *et al.* 2010). The spectrum of mutations is also identical in the three samples (Table 1). CG > TA and TA > CG transitions are the most common, with nearly equal numbers, consistent with C > T transitions arising in each sample and in the reference genome after evolutionary separation. The C > T transitions are the most frequent base change in most organisms (Lynch 2010) and are also the main component of the aging-dependent mutational signature in cancer samples (Alexandrov *et al.* 2013).

**Table 1:** All SNVs in DT40 cells and in the L2 and Silkie breeds

Base Change	DT40		L2		Silkie	
CG > AT	459,364	7.35%	455,653	7.28%	459,896	7.27%
CG > GC	445,483	7.13%	446,463	7.14%	451,513	7.13%
CG > TA	2,207,538	35.31%	2,249,362	35.96%	2,276,302	35.96%
TA > AT	485,466	7.77%	478,766	7.65%	481,672	7.61%
TA > CG	2,206,210	35.29%	2,181,601	34.87%	2,211,668	34.94%
TA > GC	447,492	7.16%	443,900	7.10%	448,589	7.09%
**Total**	**6,251,553**	**100.00%**	**6,255,745**	**100.00%**	**6,329,640**	**100.00%**

We found 1,844,901 unique SNVs in the DT40 sample, which is similar to the number of unique SNVs in the two domestic breeds used for comparison (Table 2). The mutation spectra of these unique SNV sets are different from the entire set, with an expected asymmetry appearing between CG > TA and TA > CG transitions. The increased frequency of CG > TA mutations compared with TA > CG reflects the fact that these mutations are mainly driven by C > T changes, and in the unique SNV sets we are more likely to detect changes that occurred in that particular sample, whereas in the common SNV set we are more likely to detect changes that occurred in the reference genome. The spectrum of unique SNVs also does not show any DT40-specific features. Taken together, the analysis of SNVs did not uncover any mutational processes specific to the DT40 genome.

**Table 2:** Unique SNVs in DT40 cells and in the L2 and Silkie breeds

Base Change	DT40		L2		Silkie	
CG > AT	152,918	8.28%	125,881	8.05%	153,238	8.12%
CG > GC	132,424	7.17%	112,578	7.20%	135,442	7.18%
CG > TA	733,718	39.74%	631,195	40.38%	758,788	40.23%
TA > AT	148,064	8.02%	125,262	8.01%	145,251	7.70%
TA > CG	556,837	30.16%	467,746	29.92%	572,208	30.34%
TA > GC	122,404	6.63%	100,442	6.43%	121,206	6.43%
**Total**	**1,846,365**	**100.00%**	**1,563,104**	**100.00%**	**1,886,133**	**100.00%**

We identified 708,892 indels up to 50 bp in the DT40 genome in comparison with the reference genome, which is higher than the numbers found in the L2 and Silkie breeds (Table 3). The greater number of indels in DT40 was confirmed using a different analysis tool (see *Materials and Methods*, data not shown). 62.2% of the indels were homozygous in DT40, a proportion similar to that in the domestic breeds used for comparison (Table 3). The greater number of indels in DT40 is more apparent when examining indels found in one of the three samples only (Table 4). The ratio of these unique indels to unique SNVs is higher in DT40 (0.130) than in the L2 and Silkie genomes (0.0919 and 0.0864, respectively). Considering molecular clock theory (Zuckerkandl 1987), this observation suggests that processes resulting in increased indel formation were operating in the DT40 genome. However, the proportion of heterozygous unique indels is not higher in DT40 (Table 4). As mutations arising in the cell line would be predominantly heterozygous, this suggests that the extra numbers of indels were not generated after transformation or the isolation of the cell line.

**Table 3:** All indels in DT40 cells and in the L2 and Silkie breeds

Indel	DT40		L2		Silkie	
Homozygous	441,245	62.2%	389,374	63.8%	370,100	62.3%
Heterozygous	267,647	37.8%	220,838	36.2%	223,594	37.7%
**Total**	**708,892**	**100.00%**	**610,212**	**100.00%**	**593,694**	**100.00%**

**Table 4:** Unique indels in DT40 cells and in the L2 and Silkie breeds

Indel	DT40		L2		Silkie	
Homozygous	125,420	52.3%	71,472	49.8%	78,273	48.0%
Heterozygous	114,504	47.7%	72,201	50.2%	84,728	52.0%
**Total**	**239,924**	**100.00%**	**143,673**	**100.00%**	**163,001**	**100.00%**

### CNV and LOH

**Figure 2:**
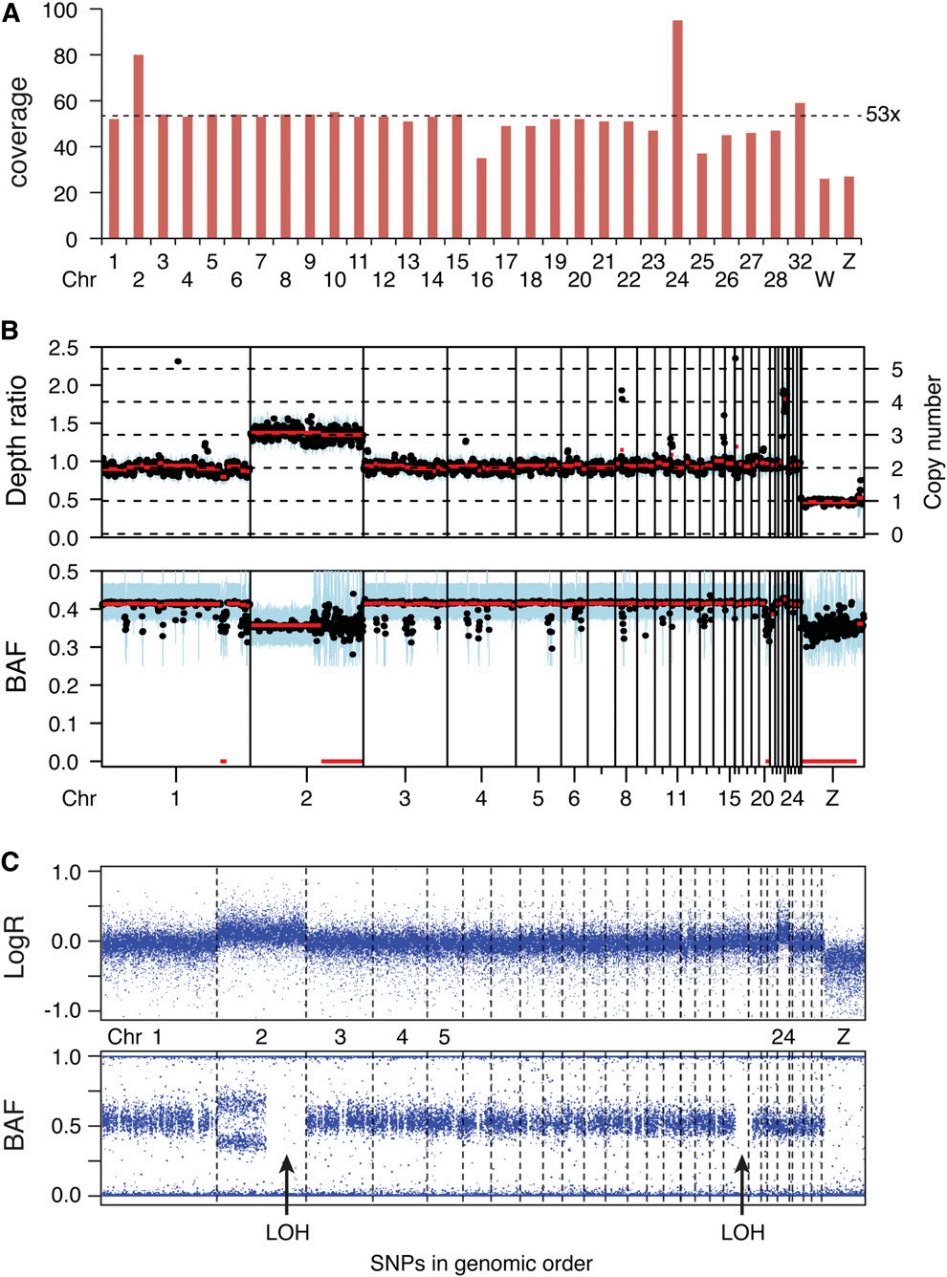
Whole chromosome copy number variation. (A) Sequence coverage of chromosomes (Chr) 1–28, 32, W, and Z. A line at 53× coverage indicates the mean coverage of larger disomic chromosomes 1, 3–10. (B) CNV and LOH analysis of the sequencing data using the Sequenza package. The plot shows the mean depth ratio and B allele frequency (BAF) of 1-Mb-sized bins overlapping every 0.5 Mb (black). The blue area represents the interquartile range of the binned data, whereas the red line indicates segments generated from 10 neighboring bins. Chromosomes 1–28, 32, W, and Z are lined-up on the X axis. The copy number scale is set according to the best fit of Sequenza’s probability model, in which the ploidy for DT40 sample was estimated to be 2.2 n. The LOH regions on chromosome 2 and 20 and whole copy number changes of chromosome 24 and 2 are visible. (C) SNP array hybridization analysis of the sequenced sample. The 60,000 SNPs are lined-up on the x axis in order of genomic occurrence. Chromosome boundaries are marked by dashed lines, and chromosome numbers are shown between the two panels. SNPs in genomic regions unassigned to chromosomes are omitted. Top panel shows signal intensity (LogR ratio, LogR); the increased copy number of chromosomes 2 and 24 is apparent. Bottom panel shows B allele frequency (BAF). Large regions of LOH are marked with arrows.

Copy number variations (CNV) are apparent from the sequence coverage of individual chromosomes (Figure 2A). The sex chromosomes W and Z are present at 26× and 27× coverage, respectively, confirming their monosomic status and the female origin of the cell line. Chromosomes 2 and 24 show much higher coverage than the expected diploid level. A number of small chromosomes deviate from the mean coverage, indicating that overall sequence coverage is not a reliable measure of ploidy for chromosomes less than approximately 5 Mb in length. It is possible that there is bias against microchromosomes in the sample preparation procedure. We also analyzed CNV using the Sequenza package (Favero *et al.* 2014), which indicated, based on read depth, that chromosome 2 is trisomic, whereas chromosome 24 is tetrasomic (Figure 2B).

For an independent measure of CNV, we analyzed the sequenced sample on a 60,000-probe SNP hybridization array (Groenen *et al.* 2011). The total signal intensity (plotted as LogR ratio) (Figure 2C) confirms the increased number of chromosomes 2 and 24. A plot of the SNP allele frequencies (B allele frequency) shows two distinct nonhomozygous states at chromosome 2, confirming its trisomic status (Figure 2C). At chromosome 24, the only intermediate allele frequency is approximately 0.5. Because the sequence coverage of this chromosome is approximately two-fold higher than that of similarly sized disomic chromosomes, this confirms that chromosome 24 is tetrasomic with two allelic variants, and the four copies do not segregate independently. Importantly, we did not see any evidence of large-scale CNV at the sub-chromosomal level.

To assess CNV within and between laboratory strains, a wild-type stock that has been widely used for the analysis of immunoglobulin diversification, Clone 18 (Buerstedde *et al.* 1990), was included in the analysis. A bulk population plus two single cell clones were analyzed from each stock; one of the WT-IAH single cell clones is the sequenced sample. In WT-CL18, there are two more trisomic chromosomes (chromosome 14 and 20); otherwise, it appears identical to WT-IAH (Figure S1). Importantly, we did not find differences between the bulk sample and two single-cell clones in either stock apart from a partial loss of heterozygosity (LOH) in chromosome 21 of the bulk WT-IAH, which is complete in the two isolated clones (Figure S1).

**Figure 3:**
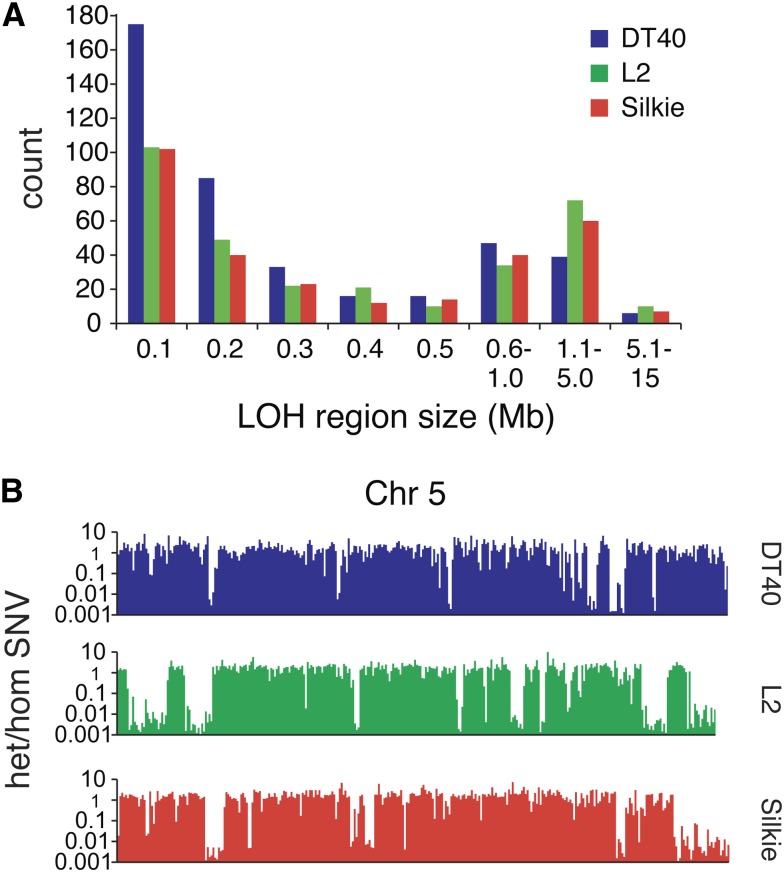
Regions of copy-neutral loss of heterozygosity. LOH regions were scored in 100-kb sequence blocks according to criteria detailed in the main text. (A) Size distribution of LOH regions in the DT40, L2, and Silkie genomes. (B) The distribution of LOH regions on chromosome (Chr) 5 of the DT40, L2, and Silkie genomes as illustrated by the ratio of heterozygous to homozygous SNVs in 100-kb sequence blocks. A ratio less than 0.1 was used to define LOH.

The SNP array shows multiple genomic regions that lack heterozygous SNPs, most notably a large part of chromosome 2 (Figure 2B). The genome sequence allows a more detailed view of regions of copy number neutral LOH (referred to simply as LOH). We calculated the ratio of heterozygous to homozygous SNVs in 100-kb sequence blocks along each chromosome and detected an average of 322 homozygous and 306 heterozygous SNVs per 100 kb; 26% of the sequence blocks have a heterozygous-to-homozygous (het/hom) ratio less than 0.1, which we classified as LOH. A further 8% contained fewer than 50 homozygous SNVs and were not used for LOH classification. In the L2 and Silkie breed samples, we classified 33% and 30% of the genome as LOH regions, respectively, indicating that there is no overall DT40-specific process resulting in large-scale LOH. The size distribution of the LOH regions is also similar between DT40 and the two domestic breeds (Figure 3A), although there is a greater incidence of short LOH regions (100–200 kb), possibly indicating higher homologous recombination activity in DT40. The size distribution suggests the presence of a larger number of LOH regions below the 100 kb size, but the SNV density does not allow a reliable detection of smaller regions. The position of the LOH regions is mostly unique in the three investigated samples, as illustrated on a selected chromosome (Figure 3B). The two largest LOH regions in the DT40 genome on chromosomes 2 and 20 were confirmed by the Sequenza analysis (Figure 2B). A table of the LOH regions in the DT40 genome at 100-kb resolution is presented as supplementary information (Table S2).

### Coding sequence changes

We investigated the mutations affecting expressed genes to gain insight into the molecular properties of DT40. The gene transcripts of the Ensembl *Gallus gallus* genebuild updated in December 2013 contained 78,329 point mutations in comparison with the reference genome. This corresponds to a divergence rate of 1.825/kb, lower than the genome-wide rate of 5.829/kb, indicative of selection against mutations.

**Figure 4:**
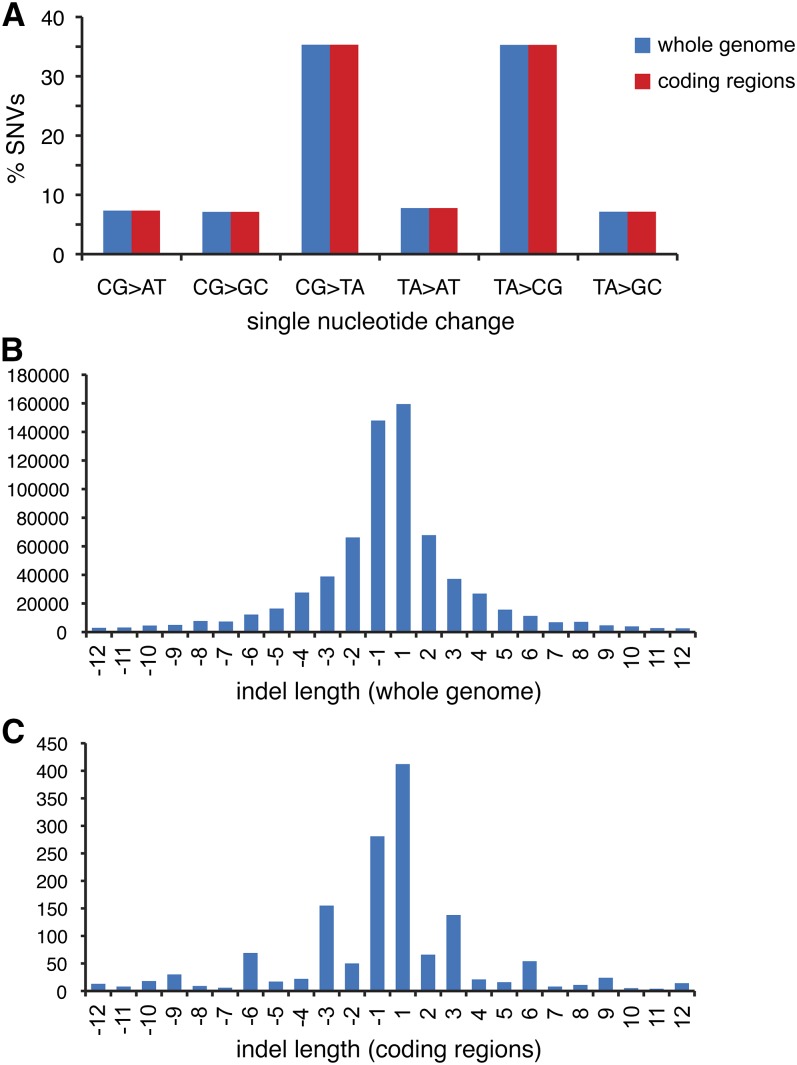
Coding region sequence alterations. Single nucleotide variations (SNVs) and insertion/deletion mutations (indels) were detected in the DT40 genome in comparison to the *Gallus gallus* reference genome. (A) Identical SNV spectrum in the coding regions of the DT40 genome and at the whole genome level. The percentage of SNVs in each of the six single nucleotide change categories is shown. (B) Length distribution of indels in the whole genome. The total number of insertions (positive) and deletions (negative) at each length is shown. (C) Length distribution of indels in coding regions, as shown in (B). The relative increase in the frequency of indels of lengths that are multiples of 3 is apparent.

The spectrum of SNVs in coding sequences is very similar to that in the whole genome (Figure 4A), suggesting that mutagenic processes affect different genomic regions indiscriminately. We further categorized these mutations using the CooVar program according to their likely disruptive effect on protein structure as based on the Grantham matrix (Grantham 1974; Vergara *et al.* 2012). The DT40 genome contains 1251 nonsynonymous mutations classified as radical, the chemically most dissimilar category of amino acid substitution. The number of radical nonsynonymous mutations is not significantly different from the 1285 and 1128 radical nonsynonymous mutations found in the L2 and Silkie breeds, respectively (Table 5). Again, DT40 does not contain more differences from the reference genome than the two domestic breeds, making it unlikely that many of these mutations arose in the cell line after its isolation.

**Table 5:** Mutations affecting protein coding regions in DT40 cells and in the L2 and Silkie breeds

Coding Region Mutation	DT40	L2	Silkie
Nonsynonymous SNVs	23,184	22,906	21,851
Unique homozygous nonsynonymous SNVs	4792	3911	4471
Radical nonsynonymous SNVs	1251	1285	1128
Unique homozygous radical nonsynonymous SNVs	242	252	241
Coding region indels	1505	1235	1074
Unique homozygous coding region indels	363	204	201
Stop-gain mutations	175	167	157
Unique homozygous stop-gain mutations	42	41	42

We also found 1505 short indels in coding exons in DT40. Most common are one-base insertions and one-base deletions. Their length distribution is similar to that of all genomic indels, but an overall pattern of selection against frameshift-causing indels is apparent (Figure 4, B and C). Following comparison with the L2 and Silkie genomes, we found 363 homozygous indels unique to DT40 (Table 5). Finally, we detected 175 nonsense mutations in DT40 that give rise to a stop codon and are therefore likely to interfere with protein function (Table 5). Further types of mutations (*e.g.*, in splice sites, stop-loss mutations) may also affect gene function. These were not considered in detail, but their numbers are presented in Table S3.

We concentrated on radical nonsynonymous mutations, indels, and stop-gain mutations in our search for genes with impaired function in DT40. We excluded those homozygous mutations that were not unique to DT40 and, taking into account that certain genes contain multiple mutations, we were left with homozygous mutations in 485 genes (Table S4). In addition to homozygous sequence changes and homozygous indels, genes may be inactivated by heteroallelic combinations of different mutations. We found 126 genes with a combination of at least two radical nonsynonymous mutations, indels, and/or stop-gain mutations in their coding exons. These are listed in Table S4. We did not attempt to resolve the haplotype of these combinations, and several may not affect each of the two alleles. The list of mutated genes in Table S4 is annotated with the human orthologs of each gene, plus gene ontology (GO) terms for the chicken and the human version.

We searched the list of potentially defective genes for the presence of genes commonly mutated in major cancer types (Kandoth *et al.* 2013) and for DNA repair genes in general. We reasoned that such genes may be inactivated specifically in DT40, and therefore may have played a role in the oncogenic process during the development of the cell line or may otherwise specifically contribute to its properties. Most notably, we found mutations in the *PIK3R1* and *ATRX* tumor suppressor genes, but not in well-known DNA repair genes.

*PIK3R1* encodes differentially spliced regulatory subunits of class I phosphatidylinositol 3-kinases. The mutation in this gene is an in-frame deletion (Z:21,510,877–21,510,882), resulting in the deletion of E450 and Y451, which are conserved in all vertebrate homologs in the Uniprot database. The structure of the protein in complex with the PIK3CA catalytic subunit has been solved (RCSB ID: 4L1B). According to this structure, these residues are located in a long coiled-coil region that connects the two SH2 domains of PIK3R1 and is responsible for the connection between the two subunits (Huang *et al.* 2007). The deletion found in DT40 and similar mutations affecting the coiled coil region can be found in the COSMIC cancer mutation database. Mutations of *PIK3R1* are present at a low prevalence in multiple types of cancer (Kandoth *et al.* 2013).

*ATRX* is a chromatin remodeling factor with various roles at genomic tandem repeat sequences (Clynes *et al.* 2013). The mutation at 4:12,803,872–12,803,873 is a two-base deletion from a long stretch of thymidines on one allele and an insertion of two thymidines on the other allele, both of which cause a frameshift that destroys the C-terminal half of the protein. We confirmed these mutations by genomic PCR and DNA sequencing (not shown). *ATRX* mutations have also been found in different types of cancer; the tumor suppressor gene is mutated in 90% of cancers that maintain their telomeres by the telomerase-independent “alternative lengthening of telomeres” (ALT) pathway (Lovejoy *et al.* 2012).

### Genome stability

**Figure 5:**
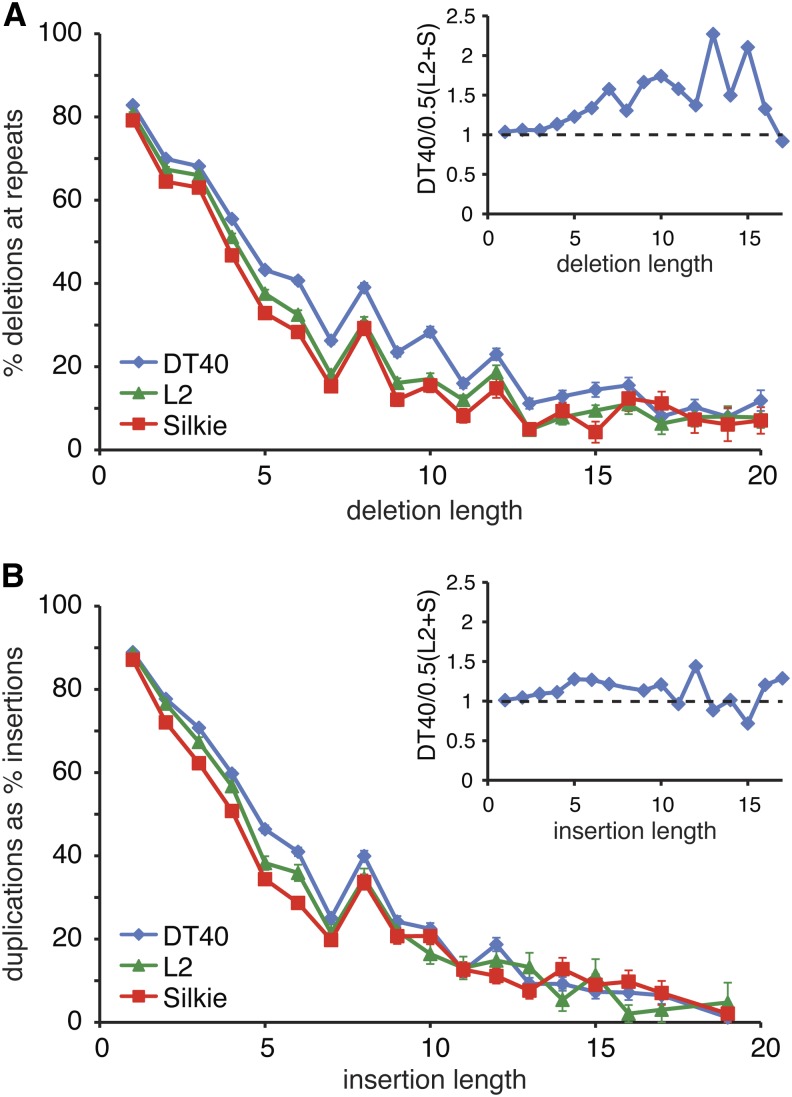
Insertion/deletion mutations at repeat sequences. (A) The percentage of deletions that precisely remove a unit of tandem repeats of at least two repeat units, plotted against the length of the deletion. Error bars are calculated as the square root of the number of events counted divided by the number of events. The inset graph shows the enrichment of the selected deletion events in the DT40 genome relative to the mean of the L2 plus Silkie (S) genomes, plotted against the length of the deletion. (B) The percentage of insertions that precisely duplicate a sequence, creating a tandem repeat, plotted against the length of the insertion. Error bars calculated as in (A). The inset graph shows the enrichment of the selected insertion events in the DT40 genome relative to the mean of the L2 plus Silkie (S) genomes, plotted against the length of the insertion.

Because DT40 is widely used to study DNA repair and damage tolerance, we asked if we see signs of genome instability that may be indicative of DNA repair defects. The only aspect by which the DT40 genome showed a noticeable difference from the L2 and Silkie chicken genomes was the number of indels (Table 3 and Table 4). Therefore, we asked if there is any evidence of repeat instability in the DT40 genome. We examined all indels that are unique to one of the three genomes (Table 4) and classified these according to whether they occurred at a repeat sequence. In case of one-nucleotide-long indels, we found no difference, with 79–82% of such deletions and 86–88% of insertions occurring at repeat sequences (Figure 5, A and B) However, longer deletions were more often found at repeat sequences in the DT40 genome than in the genome of the two domestic chicken breeds. For example, 28% of 10-base deletions are at repeats in DT40, compared with 16% and 17% in the L2 and Silkie, respectively. The enrichment of repeat-derived deletions covers the approximately 6-bp to 15-bp size range (Figure 5A). This could potentially result from a defect of mismatch repair, because MutSβ has been shown to repair insertion/deletion loops of this size range (Genschel *et al.* 1998). However, we found no verifiable mutations in the genes of mismatch repair factors. There appears to be a single base deletion causing a frameshift in the annotated exon 2 of MSH3 at Z:63,874,870. However, this mutation is in a nonconserved region of the putative chicken MSH3 protein, and EST sequences do not contain the exon; therefore, it is unlikely to be a true coding region (data not shown). We did not observe a similar increase in repeat-derived instability at insertions (Figure 5B), and the overall length distribution of indels in DT40 is not unique (Figure 4A and data not shown). Thus, we found no strong evidence of DNA repair defects shaping the DT40 genome, in agreement with the lack of mutations inactivating repair genes.

### Viral transformation

The DT40 cell line was isolated from an ALV-induced bursal lymphoma. We looked for viral insertions in the DT40 genome by performing a blastn alignment with each of the 5385 viral reference genomes present in Entrez Genomes against the *de novo* DT40 genome assembly. Of the 228 hits to scaffolds of the assembly, we excluded 14 that were short matches mainly to phages on long scaffolds that did not contain more virus sequence. The remaining 214 scaffolds contained sequences that all showed similarities to alpharetroviruses specialized to birds. The prototype of these viruses is the avian leukosis virus RSA (also known as Rous sarcoma virus), which contains three genes (gag-pro, pol, and env) between flanking LTRs. Many of the sequence hits were to the endogenous retrovirus EAV-HP, which were excluded from further analysis. The remaining scaffolds contained only viral sequences, only chicken sequences, or both. There were six scaffolds that contained both avian and viral sequences, and five of these only contained part of an LTR. Matching these scaffolds against the chicken reference genome, we found five viral integration sites. Using the independent method of mapping raw read pairs that contain a viral sequence in one of the reads did not reveal any further viral insertion sites (data not shown).

**Figure 6:**
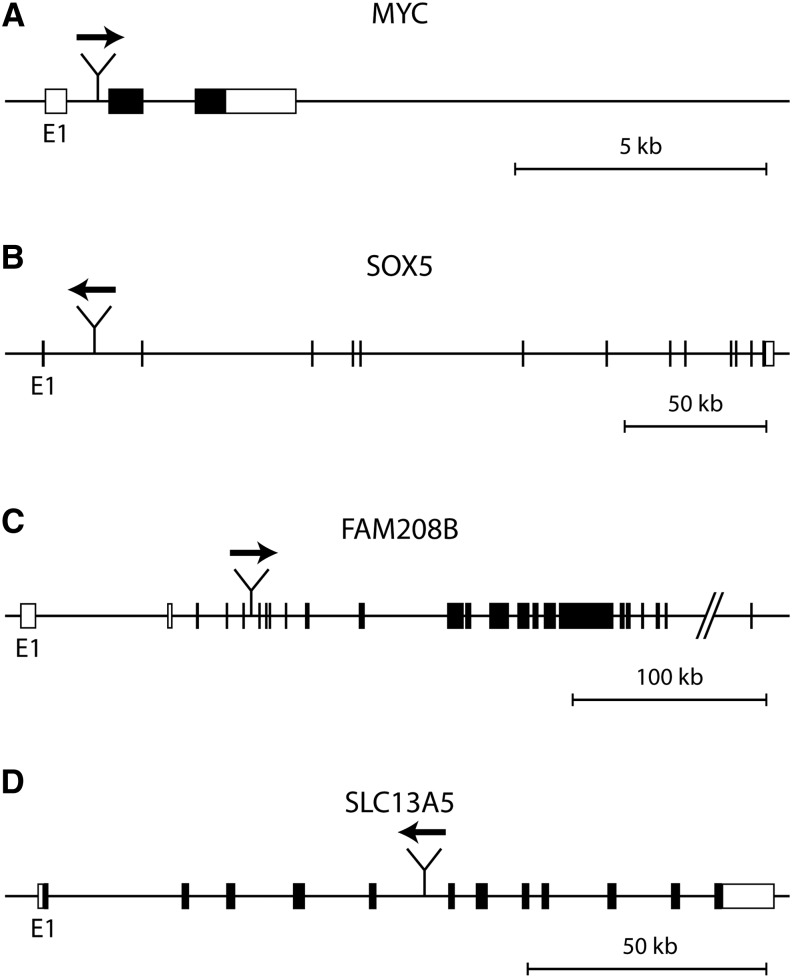
ALV insertions in the DT40 genome. Schematic drawing of ALV integration events in the DT40 genome. (A) MYC gene, (B) SOX5 gene, (C) FAM208B gene, and (D) SLC13A5 gene. The genes are transcribed left to right, with the first exon of each gene marked E1. Noncoding sequences are shown as empty boxes; coding exons are shown as filled boxes. The location and orientation of the integrated ALV copy are shown by a thick arrow above the sequence.

One of the integration sites was in the *MYC* (c-myc) locus, as expected. Rearrangement of the genomic *MYC* locus and increased *MYC* expression have been observed in the DT40 cell line (Baba *et al.* 1985), and ALV and related retroviruses have been shown to activate the cellular *MYC* proto-oncogene by proviral insertional mutagenesis (Hayward *et al.* 1981). We mapped the viral insertion to the first intron of *MYC* at the approximate position of Chr2:139,318,028 in a TA dinucleotide repeat (Figure 6A). Because the translation start site is located in the second exon of *MYC*, promoter activity of the viral long terminal repeat can activate the expression of the entire c-myc protein as reported (Hayward *et al.* 1981). The *MYC* gene is on the triploid second chromosome in the region that displays loss of heterozygosity, and the viral insertion is present in all three *MYC* alleles because there are no reads spanning the insertion site. Three further integration sites were mapped to the *SOX5*, *FAM208B*, and *SLC13A5* genes. *SOX5* encodes a high mobility group protein with a role in cell differentiation, and it has been isolated as a target of retroviral insertional mutagenesis in mouse brain tumors (Johansson *et al.* 2004). The viral insertion, which is not present in the L2 and Silkie genomes, was identified previously in white leghorn chickens as an insertion site for the ubiquitous chicken retrovirus ev1 (Hishinuma *et al.* 1981). The virus integrated in a reverse orientation relative to the *SOX5* gene at Chr1:66,086,819 (Figure 6B). It is present in both alleles in a 900-kb LOH region (Table S2). The FAM208B gene product is of unknown structure and function, and the effect of the viral integration at Chr1:1,050,550 in the putative fifth intron of one allele is unclear (Figure 6C). The integration site in the *SLC13A5* sodium-citrate cotransporter gene at Chr19:9,782,140 between the fifth and sixth exons is likely to disrupt this 12-transmembrane helix protein (Figure 6D). The last identified viral integration site is also present in the reference genome between Chr1:32,561,911–32,568,956, and it has been described as the endogenous retrovirus (ERV) locus first called JFevB (Levin *et al.* 1994).

## Discussion

The chicken DT40 cell line has been extensively used for genetic studies of DNA repair and related processes. Taking advantage of high-throughput whole genome shotgun sequencing, in this study we characterized the genomic landscape of this cell line. The relatively normal, stable karyotype together with a mutational landscape very similar to that of two domestic chicken genomes suggests the suitability of DT40 as a model for studying normal cellular processes. Additionally, the analysis of viral sequence insertions and gene mutations shed light on the oncogenic transformation of the cell line.

One of the properties that makes DT40 useful for genetic studies is its relatively normal karyotype. We compared two commonly used wild-type lines, and the main abnormality we found was the presence of extra copies of a small number of whole chromosomes. The WT-IAH stock displayed trisomy of chromosome 2 and likely tetrasomy of chromosome 24, whereas the WT-CL18 showed additional trisomy of chromosomes 14 and 20. The analysis of a bulk population and two independent clones did not reveal any copy number variability in either stock, in contrast to an earlier report that found macrochromosomal variation between and mosaicism within wild-type DT40 stocks, with only 40–66% of clones conforming to the modal karyotype concerning the five largest chromosomes (Chang and Delany 2004). The difference may be attributable to different culture conditions or a stabilization of the karyotype, although the limited number of clones in our analysis precludes direct comparison. Our results support the further use of DT40 as a model cell line for genetic studies.

We were fortunate to be able to compare the DT40 sequence to the high-quality whole genome sequence of two domestic chicken breeds obtained with the same sequencing technology (Fan *et al.* 2013). These breeds may not be the ideal comparison for the DT40 cell line derived from a commercial layer breed with a much larger likely effective population size. Nevertheless, when compared with the *Gallus gallus* reference genome, the cell line–derived sample and those from the L2 and the Silkie breeds have indistinguishable point mutation rates and mutation spectra. SNP rates in chickens have been reported to be high (Wong *et al.* 2004). The three samples showed equally high SNV rates relative to the red jungle fowl genome (5.8–5.9 SNV/kb), and the divergence between the samples is also as high as 3.0–3.5 SNV/kb. This is not a consequence of the evolutionary distance between the red jungle fowl and domestic chicken breeds, which elsewhere has been calculated as approximately 6000 years (Rubin *et al.* 2010). Instead, this gives further evidence that sequence heterogeneity is high in chicken populations in general. This phenomenon facilitates the use of techniques that rely on sequence diversity, such as CNV determination, but also potentially hinders the use of nonisogenic gene targeting constructs.

The identical spectrum of the unique SNVs in each of the three samples suggests that no specific mutational processes operate in the DT40 cell line. However, it would take a large number of extra mutations to change the mutation spectrum derived from more than 1.5 million SNVs. Therefore, this conclusion could only be drawn with certainty if a DNA sample from the DT40 source animal was available for analysis.

The high density of SNVs allowed a detailed scan of the genome for regions of LOH. Although the analysis did not reveal any DT40-specific effects, it was surprising to find that a very large proportion of the three analyzed chicken genomes had lost heterozygosity, especially because this seemed to have happened independently in the different breeds. Copy-neutral LOH may be a relatively common outcome of DNA damage tolerance processes, and selective breeding of domestic animals could make use of this source of genetic variation, increasing the proportion of the genome that is near-homozygous.

A large number of mutations were found in the coding regions of the DT40 genome. Although the overall numbers of coding region mutations do not bear evidence of DT40-specific mutational processes, it was still worth investigating if any gene mutation could be found that significantly impacted the properties of the cell line. In case of nonsynonymous point mutations, an arbitrary decision had to be made to restrict the analysis to a subset of the 4792 unique homozygous SNVs, even though mutations not classified as “radical” may also critically influence the function of particular proteins. The curated list of mutated genes made available as a supplement may contain mistakes due to the imperfections of the reference genome. For example, the analysis showed a frameshift deletion in the DNA methylase DNMT1, but on closer inspection it is likely that this "frameshift" is the consequence of the wrong annotation of a short intron in the gene sequence (data not shown). The incorrect MSH3 mutation mentioned is also the consequence of incorrect exon/intron calling.

The causes of transformation in DT40 were known to include an insertion of ALV into the c-myc gene. We mapped this insertion to the exact TA repeat location of an ALV insertion in a different bursal lymphoma precisely mapped by Westaway *et al.* (1984). Another ALV insertion was mapped 330 bp further upstream in the same study; therefore, the TA repeat insertion site is not a unique position for ALV-driven upregulation of c-myc activity. All three copies of the homozygous part of chromosome 2 contain the insertion at the same site, suggesting that both the LOH event and the copy number gain happened after the viral integration event. In addition, we also mapped further copies of ALV and related viruses using the *de novo* sequence assembly. The DT40 genome is relatively virus-free: only ALV and related chicken endogenous retroviruses were found.

In addition to ALV, we looked for additional causes of transformation among mutated genes and found the *PIK3R1* and *ATRX* mutations. The mutation of the PI3 kinase regulatory subunit is expected to contribute to the dysregulation of cell growth, as seen in many cancers. More puzzling is the *ATRX* mutation, because this is commonly found in telomerase-negative tumors that rely on the ALT pathway for telomere maintenance. However, DT40 shows a high level of telomerase expression (Swanberg and Delany 2003) and shorter telomere lengths than characteristic of ALT (O’Hare and Delany 2011). It is possible that the *ATRX* mutation contributed to the oncogenic transformation through the effect of *ATRX* on gene expression near genomic tandem repeats (Law *et al.* 2010).

Gene expression changes not detected by sequence analysis may also contribute to transformation. Currently, only a limited DT40 gene expression microarray analysis is available (Neiman *et al.* 2006). A recent study also determined the comprehensive microRNA expression profile of the DT40 cell line, finding significant and unique differences in both naïve and induced B cells (Yao *et al.* 2013). Such analyses, coupled with whole transcriptome sequencing, can shed further light on the causes of oncogenic transformation.

The DT40 cell line is considered to have a high level of homologous recombination that allows gene disruptions due to the high ratio of homologous to random integration of gene targeting constructs. Although the genome analysis did not reveal any obvious signs of high homologous recombination, the higher levels of short LOH blocks and deletions within repeats could be related to higher recombination activity.

In conclusion, our characterization found all the investigated properties of the DT40 genome to be relatively normal. With its near-normal and mostly stable karyotype, a lack of cell line–specific mutational processes and a lack of inactivating mutations in important DNA repair genes, it seems an excellent choice of cell line for continued research in the areas of DNA repair and related processes. The genome sequence, a blast database of the *de novo* assembly, and a detailed list of genes mutated in the cell line is made available to the research community. We believe that the availability of whole genome sequence data from a growing range of cell lines will greatly aid the planning and interpretation of cell line–based experiments.

## Supplementary Material

Supporting Information
