# Supplementary material for: The Genome of the Chicken DT40 Bursal Lymphoma Cell Line
Source: G3 (Bethesda). 2014 Sep 15;4(11):2231–40. doi: 10.1534/g3.114.013482 (PMC4232548; doi:10.1534/g3.114.013482)
Supplement: Supporting Information [file supp_4_11_2231__index.html]

The Genome of the Chicken DT40 Bursal Lymphoma Cell Line — Supporting Information 

# The Genome of the Chicken DT40 Bursal Lymphoma Cell Line

## Supporting Information for Molnár *et al.*, 2014

**Files in this Data Supplement:**

- Supporting Information - Figure S1, File S1, and Tables S1-S4 (PDF, 354 KB)
- Figure S1 - SNP array hybridization analysis of two wild type DT40 cell line stocks. (PDF, 333 KB)
- File S1 - Computer scripts used in data analysis. (PDF, 124 KB)
- Table S1 - De novo assembly of 100 bp paired end reads of the DT40 cell line genome. (PDF, 129 KB)
- Table S3 - Single nucleotide variations in the DT40, Taiwan L2 and Silkie genomes. (PDF, 131 KB)
- Table S2 - Regions of loss of heterozygosity in the DT40 genome. (.xls, 54 KB)
- Table S4 - Mutated genes in the DT40 genome. (.xls, 1 MB)
