# Supplementary material for: The Genome of the Chicken DT40 Bursal Lymphoma Cell Line
Source: G3 (Bethesda). 2014 Sep 15;4(11):2231–40. doi: 10.1534/g3.114.013482 (PMC4232548; doi:10.1534/g3.114.013482)
Supplement: Supporting Information [file supp_g3.114.013482_013482SI.pdf]

G3 · Genes | Genomes | Genetics

The genome of the chicken DT40 bursal lymphoma cell line

János Molnár[*], Ádám Póti[*], Orsolya Pipek[§], Marcin Krzystanek[†], Nnennaya Kanu[‡], Charles Swanton[‡], Gábor E. Tusnády*, Zoltán Szállási[†], István Csabai[§], Dávid Szüts[*,1]

[*]Institute of Enzymology, Research Centre for Natural Sciences, Hungarian Academy of Sciences, H-1117 Budapest, Hungary, [§]Department of Physics of Complex Systems, Eötvös Loránd University, H-1117 Budapest, Hungary, [†]Center for Biological Sequence Analysis, Department of Systems Biology, Technical University of Denmark, DK-2800 Lyngby, Denmark, and [‡]Cancer Research UK London Research Institute, London, WCA2 3PX, UK

[1]Corresponding author: Institute of Enzymology, Research Centre for Natural Sciences, Hungarian Academy of Sciences, Pf. 286, H-1519 Budapest, Hungary.
E-mail: szuts.david@ttk.mta.hu

DOI: 10.1534/g3.114.013482

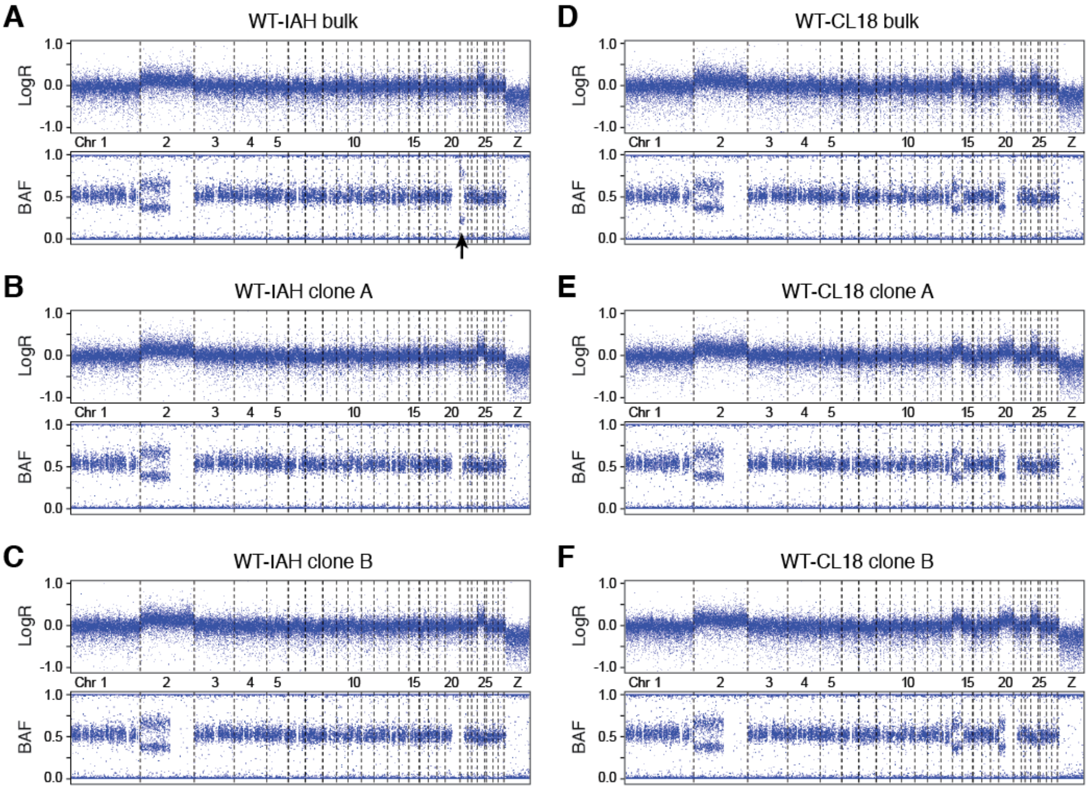

Figure S1 SNP array hybridisation analysis of two wild type DT40 cell line stocks

The 60,000 SNPs are lined up on the x axis in order of genomic occurrence. Chromosome boundaries are marked by dashed lines, chromosome numbers are shown between the two panels. SNPs in genomic regions unassigned to chromosomes are omitted. Top panel, signal intensity (LogR ratio, LogR); the increased copy number of chromosomes 2 and 24 is apparent. Bottom panel, B allele frequency (BAF). (A) a bulk population of the WT-IAH wild type line, with an arrow to point out partial loss of heterozygosity on chromosome 21. (B, C) two independent single cell clones of the WT-IAH wild type line. (D) a bulk population of the WT-CL18 wild type line. (E, F) two independent single cell clones of the WT-CL18 wild type line.

Table S1 De novo assembly of 100 bp paired end reads of the DT40 cell line genome

**Number of reads**		634926640
Contigs >=100nt	Number	263483
	Total length (nt)	**1000960390**
	Average (nt)	3798
	N50 (nt)	9330
	Median (nt)	1569
	Largest (nt)	143906
Contigs >=500nt	Number	177396
	Total length (nt)	**981176273**
	Average (nt)	5530
	N50 (nt)	9559
	Median (nt)	3326
	Largest (nt)	143906
Scaffolds >=100nt	Number	163017
	Total length (nt)	**1018805696**
	Average (nt)	6249
	N50 (nt)	25980
	Median (nt)	406
	Largest (nt)	328220
Scaffolds >=500nt	Number	77582
	Total length (nt)	**999338724**
	Average (nt)	12881
	N50 (nt)	26540
	Median (nt)	6615
	Largest (nt)	328220

Table S2   Regions of loss of heterozygosity in the DT40 genome

Table S2 is available for download at http://www.g3journal.org/lookup/suppl/doi:10.1534/g3.114.013482/-/DC1

Table S3 Single nucleotide variations in the DT40, Taiwan L2 and Silkie genomes

Mutation type	DT40	Taiwan L2	Silkie
All[1]	6251553	6255745	6329640
Intergenic[2]	3226619	3239755	3282440
Intergenic (Upstream w/in 1kb) [3]	89153	86651	83673
Intergenic (Downstream w/in 1kb) [4]	81725	83084	82473
Intergenic (Up/Down w/in 1kb) [5]	6218	6002	5469
Intronic[6]	2692379	2683657	2722589
Non-coding Intron[7]	18	4	17
Non-coding Exon[8]	895	819	894
Non-coding splicing site[9]	2	1	1
UTR5[10]	12514	11308	10419
UTR3[11]	63259	64593	64219
UTR5;UTR3[12]	73	104	66
Intron splicing site[13]	369	382	363
Exon splicing site[14]	1174	1172	1124
Coding exon[15]	78329	79385	77017
Synonymous[16]	54916	56238	54962
Nonsynonymous[17]	23184	22906	21851
Stop Gain[18]	175	167	157
Stop Loss[19]	27	29	29
Unknown[20]	27	45	18

[1]all filtered variants
[2]variant in intergenic region
[3]variant overlaps 1-kb region upstream of transcription start site
[4]variant overlaps 1-kb region downtream of transcription end site
[5]variant overlaps a gene
[6]variant overlaps an intron
[7]variant overlaps a transcript without coding annotation in the gene definition and overlaps an intron
[8]variant overlaps a transcript without coding annotation in the gene definition and overlaps the exonic region
[9]variant overlaps a transcript without coding annotation in the gene definition and variant is within 2-bp of a splicing junction
[10]variant overlaps a 5' untranslated region
[11]variant overlaps a 3' untranslated region
[12]variant overlaps a 5' untranslated region and a 3' untranslated region (e.g overlapping transcripts)
[13]variant overlaps an exon
[14]variant is within 2-bp of a splicing junction
[15]variant overlaps coding sequence
[16]variant is a silent mutation
[17]variant changes amino acid sequence
[18]variant is a nonsense mutation
[19]variant changes stop codon
[20]variant is in an incomplete ORF

Table S4 Mutated genes in the DT40 genome

Table S4 is available for download at http://www.g3journal.org/lookup/suppl/doi:10.1534/g3.114.013482/-/DC1

File S1

Computer scripts used in data analysis

File S1 is available for download at http://www.g3journal.org/lookup/suppl/doi:10.1534/g3.114.013482/-/DC1
