# Supplementary material for: The Genome of the Chicken DT40 Bursal Lymphoma Cell Line
Source: G3 (Bethesda). 2014 Sep 15;4(11):2231–40. doi: 10.1534/g3.114.013482 (PMC4232548; doi:10.1534/g3.114.013482)
Supplement: Supporting Information [file supp_g3.114.013482_FigureS1.pdf]

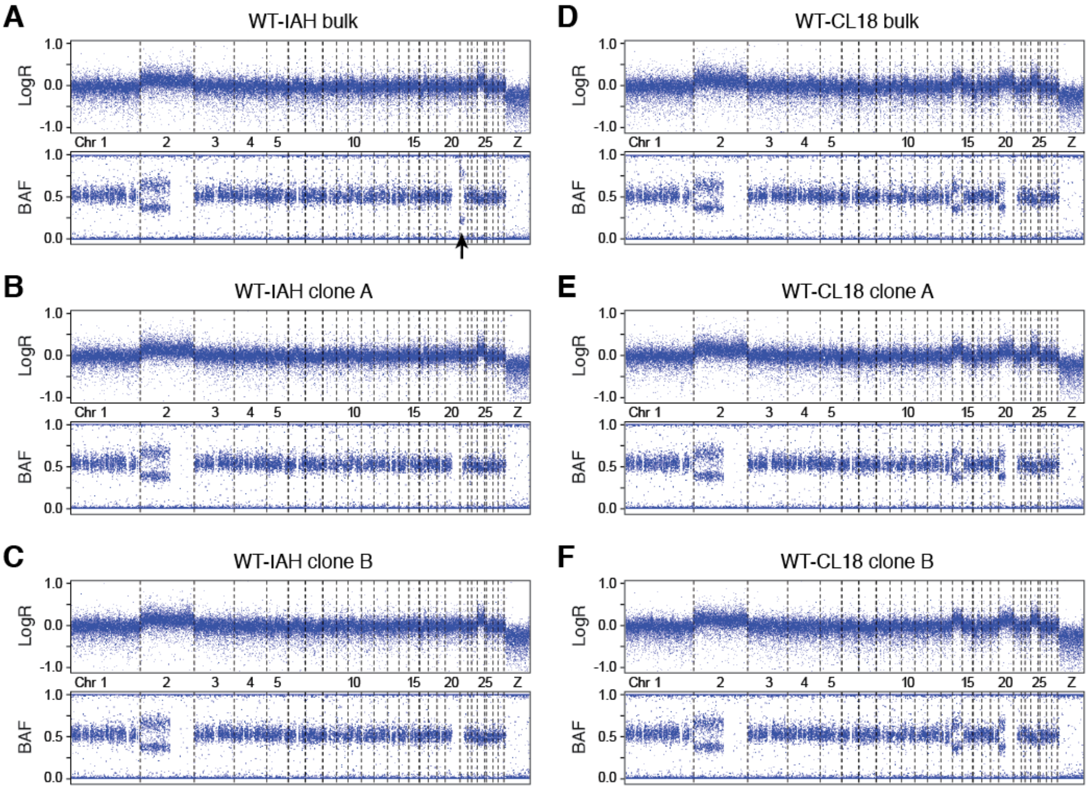

Figure S1 SNP array hybridisation analysis of two wild type DT40 cell line stocks

The 60,000 SNPs are lined up on the x axis in order of genomic occurrence. Chromosome boundaries are marked by dashed lines, chromosome numbers are shown between the two panels. SNPs in genomic regions unassigned to chromosomes are omitted. Top panel, signal intensity (LogR ratio, LogR); the increased copy number of chromosomes 2 and 24 is apparent. Bottom panel, B allele frequency (BAF). (A) a bulk population of the WT-IAH wild type line, with an arrow to point out partial loss of heterozygosity on chromosome 21. (B, C) two independent single cell clones of the WT-IAH wild type line. (D) a bulk population of the WT-CL18 wild type line. (E, F) two independent single cell clones of the WT-CL18 wild type line.
