# Supplementary material for: The Genome of the Chicken DT40 Bursal Lymphoma Cell Line
Source: G3 (Bethesda). 2014 Sep 15;4(11):2231–40. doi: 10.1534/g3.114.013482 (PMC4232548; doi:10.1534/g3.114.013482)
Supplement: Supporting Information [file supp_g3.114.013482_FileS1.pdf]

Supporting information

File S1

The genome of the chicken DT40 bursal lymphoma cell line

János Molnár*, Ádám Póti*, Orsolya Pipek§, Marcin Krzystanek†, Nnennaya Kanu‡, Charles Swanton‡, Gábor E. Tusnády*, Zoltán Szállási†, István Csabai§, Dávid Szüts*,[1]

*Institute of Enzymology, Research Centre for Natural Sciences, Hungarian Academy of Sciences, H-1117 Budapest, Hungary, §Department of Physics of Complex Systems, Eötvös Loránd University, H-1117 Budapest, Hungary, †Center for Biological Sequence Analysis, Department of Systems Biology, Technical University of Denmark, DK-2800 Lyngby, Denmark, and ‡Cancer Research UK London Research Institute, London, WCA2 3PX, UK

[1]Corresponding author: Institute of Enzymology, Research Centre for Natural Sciences, Hungarian Academy of Sciences, Pf. 286, H-1519 Budapest, Hungary. E-mail: szuts.david@ttk.mta.hu

File S1

Computer scripts used in data analysis

Reference Mapping Script:

```
#!/bin/sh
# how to run the script:
# nohup ./Reference_mapping.sh database database_index forward reverse
threads resultsdir result_file_name > name.log 2> name.err &

database=$1            # reference sequence in fasta format, complete path
database_index=$2      # reference index file, fai, complete path
forward=$3             # forward genomic sequence in fastq.gz, complete path
reverse=$4             # reverse genomic sequence in fastq.gz, complete path
threads=$5             # number of threads to run bwa
resultsdir=$6          # where to write the results
result_file_name=$7    # results file name, without file name extension

forward_name=$(basename $forward)
reverse_name=$(basename $reverse)

echo "===================================="
echo "cd $resultsdir"
cd $resultsdir

date
#--------------------------------
echo "--------------------------------"
echo "bwa aln -t $threads $database $forward > ${forward_name}.sai 2>>
${forward_name}.err"
bwa aln -t $threads $database $forward > ${forward_name}.sai 2>>
${forward_name}.err
#--------------------------------
date

#--------------------------------
echo "--------------------------------"
echo "bwa aln -t $threads $database $reverse > ${reverse_name}.sai 2>>
${reverse_name}.err"
bwa aln -t $threads $database $reverse > ${reverse_name}.sai 2>>
${reverse_name}.err
#--------------------------------
date

#--------------------------------
echo "--------------------------------"
echo "bwa sampe $database ${forward_name}.sai ${reverse_name}.sai $forward
$reverse \
| samtools view -buS -t $database_index - \
| samtools sort -m 5000000000 - ${resultsdir}${result_file_name}"
```

```
(bwa sampe $database ${forward_name}.sai ${reverse_name}.sai $forward
$reverse \
| samtools view -buS -t $database_index - \
| samtools sort -m 5000000000 - ${resultsdir}${result_file_name}) 2>>
${forward_name}_${reverse_name}.aln.err

date
echo "samtools index ${resultsdir}${result_file_name}.bam"
samtools index ${resultsdir}${result_file_name}.bam
#--------------------------------
date
echo "===================================="
```

Variant Calling Script:

```
#!/bin/bash
# how to run the script:
# nohup ./Variant_calling.sh reference bam_file output > output.log 2> output.err

reference=$1  # the reference sequence file in fasta format
bam_file=$2              # the reference mapped reads in BAM format
output=$3                # the output file name, without file extension


echo
"=================================================================
========"
date
echo "samtools mpileup -f $reference -E -D -S -u $bam_file | bcftools view -bvcg -
> ${output}.raw.bcf"
samtools mpileup -f $reference -E -D -S -u $bam_file | bcftools view -bvcg - >
${output}.raw.bcf
echo "--------------------------------------------------------------------"

echo "bcftools view ${output}.raw.bcf | vcfutils.pl varFilter > ${output}.vcf"
bcftools view ${output}.raw.bcf | vcfutils.pl varFilter > ${output}.vcf
echo "--------------------------------------------------------------------"

echo "bgzip -c ${output}.vcf > ${output}.vcf.gz"
bgzip -c ${output}.vcf > ${output}.vcf.gz
echo "--------------------------------------------------------------------"

echo "tabix -p vcf ${output}.vcf.gz"
tabix -p vcf ${output}.vcf.gz
date
echo
"=================================================================
========"
```

Insert size distribution script:

```
#!/bin/sh
# insert_size_count is a cpp program, downloaded from
https://www.biostars.org/p/14339/
# how to run the script:
# nohup ./Insert_size.sh bam_file results_directory &

$bam_file=$1
$results_dir=$2

./insert_size_count $bam_file $results_dir
```

Coverage calculation script:

1. Shell script to run the perl script

```
#!/bin/sh
# how to run the script:
# nohup ./Coverage_calc.sh bam_file > coverage.txt 2> coverage.log &

bam_file=$1

for chr in $(samtools idxstats $bam_file | awk '{print $1}'| sed '/\*/d');do
	echo -n "${chr} "
	echo "samtools view -b $bam_file $chr | bamtools coverage | awk '{print
$3}' | sed '/^0/ d' | ./Coverage_calculator.pl" >&2
	samtools view -b $bam_file $chr | bamtools coverage | awk '{print $3}' |
sed '/^0/ d' | ./Coverage_calculator.pl
done
```

2. Perl script to calculate average and median coverage

```
#!/usr/bin/perl -w
# Calculate average and median coverage
# How to run the script:
# bamtools coverage -in bam_file | awk '{print $3}' | sed '/^0/ d'|
./Coverage_calculator.pl > output 2> error


sub median {
@_ == 1 or die ('Sub usage: $median = median(\@array);');
my ($array_ref) = @_;
my $count = scalar @$array_ref;
# Sort a COPY of the array, leaving the original untouched
my @array = sort { $a <=> $b } @$array_ref;
if ($count % 2) {
return $array[int($count/2)];
} else {
return ($array[$count/2] + $array[$count/2 - 1]) / 2;
}
}


sub average {
@_ == 1 or die ('Sub usage: $average = average(\@array);');
my ($array_ref) = @_;
my $sum;
my $count = scalar @$array_ref;
```

```
foreach (@$array_ref) { $sum += $_; }
return $sum / $count;
}



while(<>)
{
	chomp;
	$input_coverage =$_;
	push(@coverage,$input_coverage);
}

$coverage_median = median(\@coverage);
$coverage_average = average(\@coverage);

print "AVG:$coverage_average\tMEDIAN:$coverage_median\n";

exit 0;
```

De novo assembly script:

```
# De novo genome assembly of the DT40 sample

#$ -V
#$ -N denovo

#$ -S /bin/bash
#$ -e name_of_the_error_file.err
#$ -o name_of_the_log_file.log

#$ -pe orte 400
echo "Starting..."
date

results_dir=full_path_to_the_result_directory
forward=full_path_to_the_DT40_R1_fastq.gz_file
reverse=full_path_to_the_DT40_R2_fastq.gz_file
Ray=full_path_to_the_RAY_binary_file

echo "mpiexec -n 400 ${Ray} -k 31 -p ${forward} ${reverse} -o ${results_dir}"
mpiexec -n 400 ${Ray} -k 31 -p ${forward} ${reverse} -o ${results_dir}

date
echo "The End"
```

Steps of LOH analysis:

1. Generating SNPDensities for Homozygous and Heterozygous SNPs

```
nohup vcftools --vcf Sample_filtered_HOM_SNPs.vcf --SNPdensity 100000 --out Sample_filtered_HOM_SNPs.100k &
nohup vcftools --vcf Sample_filtered_HET_SNPs.vcf --SNPdensity 100000 --out Sample_filtered_HET_SNPs.100k &
```

---

2. Paste the Homozygous and Heterozygous SNP numbers into One file

```
paste Sample_filtered_HOM_SNPs.100k Sample_filtered_HET_SNPs.100k > Sample.HOM.HET.100k.snpdensity
```

---

3. Selection of those regions where the number of homozygous SNVs was at least ten times greater than the number of heterozygous SNVs, and the number of homozygous SNVs was greater than 50

```
awk '{if($3>50) print $0}' Sample.HOM.HET.100k.snpdensity | awk '{if($7*10 < $3) print $0}' | grep "^[0-9]" > Sample.HOM.HET.100k.filtered.snpdensity
```

---

4. Generating Bed file and merging the neighbouring coordinates

```
awk '{print $1"\t"$2"\t"$2+100000}' Sample.HOM.HET.100k.filtered.snpdensity > Sample.HOM.HET.100k.filtered.snpdensity.bed
bedtools merge -i Sample.HOM.HET.100k.filtered.snpdensity.bed > Sample.HOM.HET.100k.filtered.snpdensity.merged.bed
```

---

5. Counting LOH length

```
awk '{print $3-$2}' Sample.HOM.HET.100k.filtered.snpdensity.merged.bed > Sample.HOM.HET.100k.filtered.snpdensity.merged.length
```

---

6. Counting the distribution of LOH length

```
for i in $(sort -h -u Sample.HOM.HET.100k.filtered.snpdensity.merged.length); do number=$(awk '{if($1=="'"$i"'") print $0}' Sample.HOM.HET.100k.filtered.snpdensity.merged.length | wc -l); echo -e -n "$i\t$number\n" ;done > Sample.HOM.HET.100k.filtered.snpdensity.merged.length.dist.txt
```
