# Supplementary material for: The Genome of the Chicken DT40 Bursal Lymphoma Cell Line
Source: G3 (Bethesda). 2014 Sep 15;4(11):2231–40. doi: 10.1534/g3.114.013482 (PMC4232548; doi:10.1534/g3.114.013482)
Supplement: Supporting Information [file supp_g3.114.013482_TableS1.pdf]

Table S1 De novo assembly of 100 bp paired end reads of the DT40 cell line genome

**Number of reads**		634926640
Contigs >=100nt	Number	263483
	Total length (nt)	**1000960390**
	Average (nt)	3798
	N50 (nt)	9330
	Median (nt)	1569
	Largest (nt)	143906
Contigs >=500nt	Number	177396
	Total length (nt)	**981176273**
	Average (nt)	5530
	N50 (nt)	9559
	Median (nt)	3326
	Largest (nt)	143906
Scaffolds >=100nt	Number	163017
	Total length (nt)	**1018805696**
	Average (nt)	6249
	N50 (nt)	25980
	Median (nt)	406
	Largest (nt)	328220
Scaffolds >=500nt	Number	77582
	Total length (nt)	**999338724**
	Average (nt)	12881
	N50 (nt)	26540
	Median (nt)	6615
	Largest (nt)	328220
