# Supplementary material for: The Genome of the Chicken DT40 Bursal Lymphoma Cell Line
Source: G3 (Bethesda). 2014 Sep 15;4(11):2231–40. doi: 10.1534/g3.114.013482 (PMC4232548; doi:10.1534/g3.114.013482)
Supplement: Supporting Information [file supp_g3.114.013482_TableS3.pdf]

Table S3 Single nucleotide variations in the DT40, Taiwan L2 and Silkie genomes

Mutation type	DT40	Taiwan L2	Silkie
All[1]	6251553	6255745	6329640
Intergenic[2]	3226619	3239755	3282440
Intergenic (Upstream w/in 1kb) [3]	89153	86651	83673
Intergenic (Downstream w/in 1kb) [4]	81725	83084	82473
Intergenic (Up/Down w/in 1kb) [5]	6218	6002	5469
Intronic[6]	2692379	2683657	2722589
Non-coding Intron[7]	18	4	17
Non-coding Exon[8]	895	819	894
Non-coding splicing site[9]	2	1	1
UTR5[10]	12514	11308	10419
UTR3[11]	63259	64593	64219
UTR5;UTR3[12]	73	104	66
Intron splicing site[13]	369	382	363
Exon splicing site[14]	1174	1172	1124
Coding exon[15]	78329	79385	77017
Synonymous[16]	54916	56238	54962
Nonsynonymous[17]	23184	22906	21851
Stop Gain[18]	175	167	157
Stop Loss[19]	27	29	29
Unknown[20]	27	45	18

[1]all filtered variants
[2]variant in intergenic region
[3]variant overlaps 1-kb region upstream of transcription start site
[4]variant overlaps 1-kb region downtream of transcription end site
[5]variant overlaps a gene
[6]variant overlaps an intron
[7]variant overlaps a transcript without coding annotation in the gene definition and overlaps an intron
[8]variant overlaps a transcript without coding annotation in the gene definition and overlaps the exonic region
[9]variant overlaps a transcript without coding annotation in the gene definition and variant is within 2-bp of a splicing junction
[10]variant overlaps a 5' untranslated region
[11]variant overlaps a 3' untranslated region
[12]variant overlaps a 5' untranslated region and a 3' untranslated region (e.g overlapping transcripts)
[13]variant overlaps an exon
[14]variant is within 2-bp of a splicing junction
[15]variant overlaps coding sequence
[16]variant is a silent mutation
[17]variant changes amino acid sequence
[18]variant is a nonsense mutation
[19]variant changes stop codon
[20]variant is in an incomplete ORF
